# Complete Genome Sequence Analysis of the First Imported Mpox Virus Clade Ib Variant in China

**DOI:** 10.3390/pathogens14010102

**Published:** 2025-01-20

**Authors:** Yin Song, Yong Yan, Jingyu Xu, Shencong Lv, Ganglin Ren, Yamei Zhou, Wanchen Song, Rui Ge, Peihua Xu, Guoying Zhu, Zhongwen Chen

**Affiliations:** 1 Jiaxing Key Laboratory of Pathogenic Microbiology, Jiaxing Center for Disease Control and Prevention, Jiaxing 314050, China; songyin1995@163.com (Y.S.); 13567309672@139.com (Y.Y.); thestorm2008@126.com (S.L.); renganglin1997@163.com (G.R.); zhouyamei168@163.com (Y.Z.); swanglebeta@gmail.com (W.S.); jxaids@163.com (R.G.); 2 Department of Microbiology, Haiyan Center for Disease Control and Prevention, Haiyan, Jiaxing 314300, China; 13857327399@139.com (J.X.); rambow_hy@outlook.com (P.X.)

**Keywords:** mpox virus, whole genome sequencing, phylogenetic analysis, gene mutation analysis

## Abstract

Mpox, a zoonotic disease caused by the mpox virus (MPXV), has seen a significant shift in its epidemiological status since 2022, evolving from an initial local outbreak to a global epidemic. This recent outbreak of MPXV mainly emerged in several European and American countries and subsequently spread to over 100 countries and regions worldwide. The rapid evolution of MPXV, coupled with increased international interactions, has led to a gradual rise in mpox cases in certain regions of Asia, mostly involving MPXV clade II and its branch strains. In contrast, the more pathogenic and clinically severe MPXV clade Ib has been relatively rare, with no reports in China to date. Here, we analyzed the whole gene sequence of imported MPXV clade Ib variant from the first infection case detected in China. Through whole genome sequencing, we successfully obtained a full-length MPXV genome of 195,405 base pairs (bp). Phylogenetic analysis revealed that the genetic sequence of the MPXV in this case was predominantly clustered with MPXV clade Ib sequences previously reported from multiple African and European countries. Compared with the MPXV clade Ib reference strain DQ011155.1, there are 127 nucleotide alterations and 57 amino acid mutations in the MPXV genome of this case. Given that the MPXV clade Ib has started to appear in China, we must pay more attention to the prevention of and control measures for the spread of mpox.

## 1. Introduction

Mpox, a zoonotic infectious disease caused by the MPXV, has the capacity to infect both humans and animals [1,2]. The virus was first identified in monkeys in 1958 and the first human case of mpox infection was diagnosed in the Republic of Congo, Central Africa, in 1970, marking the beginning of its discovery in a diverse range of animal species [3,4,5]. Since then, mpox has predominantly circulated in Central and West Africa, with transmission occurring through animal-to-human, animal-to-animal, and human-to-human contact [6,7,8]. The most recent outbreak, which began in 2022, has escalated into a global mpox pandemic, impacting up to 110 countries and regions worldwide [9]. The rapid evolution of the virus, coupled with the increase in international travel, has led to a rise in mpox cases in certain regions of Asia [10,11,12].

MPXV, a member of the family Poxviridae and the genus Orthopoxvirus, is characterized by a brick- or oval-shaped morphology with a diameter of approximately 200–250 nm [13,14]. Its genome comprises a linear double-stranded DNA of about 197 kb, encoding for approximately 180 proteins [15]. The virus is classified into two distinct evolutionary clades: clade I, which includes clade Ia and Ib and was previously referred to as the Central African or Congo Basin clade; and clade II, which includes clade IIa and IIb and was known as the West African clade [16]. The strain of MPXV circulating during the 2022 outbreak is attributed to the B.1 lineage of the clade IIb. The MPXV strains isolated since 2022 have displayed more mutations in genes possibly associated with virulence, host recognition, and immune evasion [17,18,19]. While the functional implications of these mutations are not yet fully comprehended, the elevated mutation rate may account for the MPXV’s increased prevalence in non-endemic regions.

High-risk groups for mpox are primarily composed of healthcare workers who are in direct contact with the virus, individuals who have close contact with mpox patients, children, pregnant women, and men who have sex with men. In recent years, the acceleration of globalization, enhanced population mobility, and the expansion of trade networks have facilitated the international dissemination of mpox, resulting in outbreaks across multiple countries globally [20,21,22]. As the number of infected patients continues to rise, the significance of vigilance and attention to mpox has become more pronounced [23].

## 2. Materials and Methods

### 2.1. Ethical Statement

Informed consent was obtained from the participant before this study was conducted. The study and all procedures were approved by Jiaxing Municipal Center for Disease Control and Prevention (Approval code: 2024-02) and carried out in accordance with biosafety and ethical standards of the institutional and national research committee and the relevant laws and regulations of People’s Republic of China.

### 2.2. Real-Time PCR

Viral DNA was extracted from biological samples using an EXM6000 nucleic acid extractor (Zybio, Chongqing, China) with the corresponding extraction kit (Zybio RB-T 200, Lot.2411002). Subsequently, real-time PCR experiments were performed to detect MPXV nucleic acid using the mpox virus nucleic acid detection kit (x-abt D2361YH, Beijing, China). All procedural steps are strictly adhered to as delineated in the respective kit manuals to ensure the accuracy and reliability of the experimental outcomes.

### 2.3. Whole Genome Sequencing

Viral DNA was extracted from clinical samples using the QIAamp DNA Kit (Qiagen, Hilden, Germany, catalog number 51306). Subsequently, the MPXV whole genome was enriched from the extracted viral DNA using the Target Capture Kit for Mpox Virus (Baiyi Technology Co., Ltd., Hangzhou, China, catalog number BK-MPXY024). For Nanopore sequencing, a sequencing library was constructed in accordance with the manufacturer’s protocols provided with the Sequencing Ligation Kit (Oxford Nanopore Technologies, Oxford, UK, catalog number SQK-LSK114) and the library building kit (Baiyi Technology Co., Ltd., Hangzhou, China, catalog number BK-AUXS008). Following quantitative dilution, the library was loaded onto the flow cell R10.4.1 (Oxford Nanopore Technologies, Oxford, UK, catalog number FLO-PRO114M) and sequenced using the P2 Solo protocol (Oxford Nanopore Technologies, Oxford, UK). Post-sequencing data analysis was performed utilizing the MPXY module within the BAIYIMicroGeno Platform (version 5.2, Hangzhou Baiyi Technology Co., Ltd., Hangzhou, China).

### 2.4. Phylogenetic Analysis

We constructed phylogenetic trees by combining the virus genome sequence from case samples with genome sequences submitted by different institutions to GISAID and NCBI at different times, in order to identify the evolutionary lineage to which the case’s strain belongs. Phylogenetic tree analysis was conducted using the Maximal likelihood method with IQ-TREE (version 2.0.3, available at https://github.com/iqtree/iqtree2, accessed on 1 January 2025). The results were handled and visualized with iTOL (https://itol.embl.de/, accessed on 1 January 2025).

## 3. Results

### 3.1. Case Diagnosis

On 30 December 2024, the People’s Hospital of Haiyan County, China, reported a suspected case of mpox, which was a 28-year-old South African female. On the same day, the specimens were sent to the HaiYan Center for Disease Control and Prevention for further review, and the case was ultimately confirmed to be infected with MPXV by real-time PCR. On 31 December, the Jiaxing Center for Disease Control and Prevention promptly performed whole genome sequencing using the Nanopore platform (Oxford Nanopore Technologies, Oxford, UK, GridION X5). Evolutionary analysis of the sequencing data confirmed the case’s infection with the MPXV clade Ib. This diagnosis was subsequently validated by sequencing results obtained from the Zhejiang Provincial Center for Disease Control and Prevention.

### 3.2. Detection of Mpox Virus

In total, 13 biological samples were collected from various parts of this case’s body, including the respiratory tract, limbs, face, blood, and so on. Real-time PCR was employed to detect the gene fragment Ct-*F3L* of the MPXV. The nucleic acid detection results from 12 different parts of the case’s body were all positive for the MPXV. The lowest *C_T_* values were observed in the herpes fluid, ranging from approximately 18 to 20. The values for the facial herpes fluid and throat swabs were slightly higher, between approximately 26 and 27. Notably, the highest *C_T_* value was detected in the blood sample, which was 34.08. For more detailed information, refer to Table 1.

### 3.3. Analysis of Phylogenetic Tree

**Figure 1:**
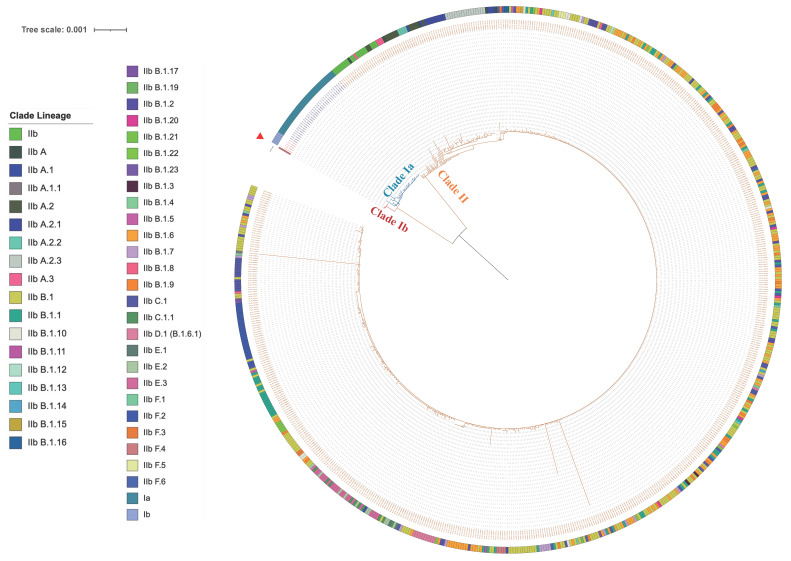
Phylogenetic tree analysis of all the selected MPXV sequences from GISAID. The red branch represents the Clade Ib sequences, the blue branch represents the Clade Ia sequences, and the orange branch represents the Clade II sequences. The red symbol “▶” denotes the sequence hMpxV/China/JXHY/2024/12.

Using Nanopore sequencing technology, we obtained the complete genome of MPXV from the sample of arm herpes, which is 195,405 bp in length, and designated the strain as hMpxV/China/JXHY/2024/12. Phylogenetic analysis revealed that the virus sequence was closely related to MPVX clade Ib sequences (Figure 1). This result was also confirmed by the phylogenetic tree constructed using the GISAID and NCBI Blast tools, indicating that the strain from the case indeed belongs to MPVX clade Ib.

**Figure 2:**
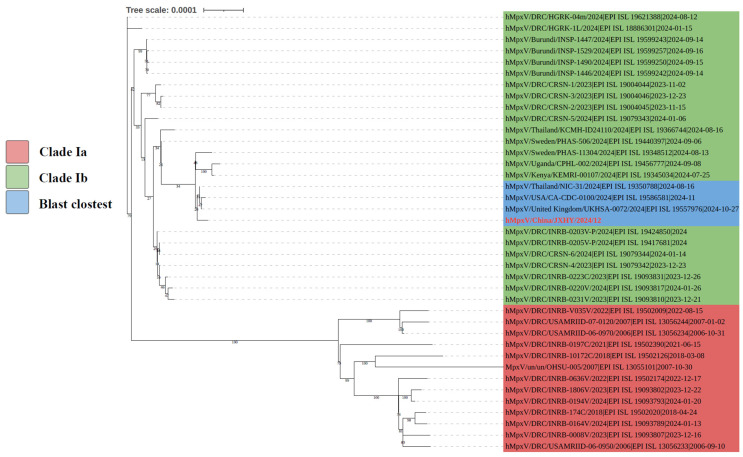
Phylogenetic tree of the 25 MPXV clade I sequences with hMpxV/China/JXHY/2024/12. The red area represents the MPXV clade Ia sequences, the green area represents the MPXV clade Ib sequences, and the blue area represents the sequence with the closest similarity. The red font text represents the sequence of the MPXV that we isolated.

BLAST analysis of the sequence hMpxV/China/JXHY/2024/12 against the MPXV sequences in the GSAID database showed that it shares the highest similarity (99.978%) with the sequence hMpxV/United_Kingdom/UKHSA-0072/2024. Furthermore, the majority of sequences closely related to hMpxV/China/JXHY/2024/12 are from African countries, including Burundi, Democratic Republic of Congo (DRC), Kenya, Uganda, etc. Among the top 25 similar sequences, there are also MPXV sequences from Sweden, the USA, and Thailand. For further phylogenetic analysis, we selected the top 25 similar sequences (Figure 2).

### 3.4. Analysis of Mutation Sites

Compared to the MPXV strain DQ011155.1 (Monkeypox virus strain Zaire_1979-005, complete genome), our analysis identified 127 nucleotide (Table S1) and 57 amino acid sites of missense mutations within the genome of hMpxV/China/JXHY/2024/12 (Table 2).

## 4. Discussion

The MPXV has distinct characteristics in its distribution within human tissues. MPXV gains entry into nearby tissues through mucous membranes, such as the conjunctiva, respiratory tract, mouth, urethra, and rectum, or through broken skin, following contact with respiratory secretions or body fluids from a patient with mpox [24,25,26]. The virus then disseminates throughout the body, leading to lymph node enlargement and the onset of a rash that initially appears on the head and face before spreading to the rest of the body. The rash progresses from papules to herpes and pustules, culminating in healing crusts that often leave scars [27,28]. In our detection results, the highest MPXV content was detected in the herpes fluid from the back and lower limbs, followed by the herpes fluid from the face and the oropharyngeal swab, with the lowest concentration found in the blood (Table 1). Despite variations in viral content between herpes fluid and oropharyngeal swabs, both showed high viral loads. In contrast, the MPXV content in the blood was significantly lower than that in other samples, aligning with the characteristics of viruses transmitted through close contact and causing localized symptoms, which is markedly different from blood-borne viruses such as HIV. Consequently, when collecting specimens for such cases, priority should be given to herpes fluid, mucosal secretions from the throat or anus, with blood collection considered as a secondary option.

Previously, mpox cases in China have predominantly been caused by MPXV clade II, aligning with the current global circulating strain [29,30]. The MPXV strain hMpxV/China/JXHY/2024/12, reported here, clustered with clade Ib and was the first detection of the MPXV clade Ib in China. Phylogenetic analysis with MPXV strains in GSAID revealed that it was closely related to sequences from the UK, Sweden, the USA, Thailand, and several African countries (Burundi, DRC, Kenya, and Uganda), with the highest similarity to a UK strain, hMpxV/United_Kingdom/UKHSA-0072/2024|EPI_ISL _19557976|2024-10-27 (Figure 2). Additionally, BLAST analysis with MPXV sequences in NCBI showed that it had most similarity (Query cover 100%, Per. ident 99.98%) with Thailand strain (Monkeypox virus isolate Monkeypox virus/Human/Thailand/DDCBIDI-002/2024, partial genome), Germany strain (Monkeypox virus isolate MPXV/Ib/Germany/2024/RK11142, partial genome) and the UK strain (Monkeypox virus isolate MPXV_UK_2024_0072, partial genome) [31]. The carrier of the Thailand strain reported that he was a European in Thailand who had a history of travel to Africa. Coincidentally, the case we analyzed also originated from South Africa, and hMpxV/China/JXHY/2024/12 clustered with multiple African MPXV clade Ib strains. This suggested that the virus transmission possibly originated from Africa. However, the case claimed not to have left China in the past four months, but traveled to Guangzhou on 15 December 2024, to meet with companions. Given that the incubation period for mpox typically ranges from 6 to 13 days, the case may have been infected with the virus through close contact with individuals from Europe or Africa residing in Guangzhou. Although the case did not mention other regions, the possibility of infection during their journey cannot be excluded. The MPXV clade Ib can be transmitted not only through close contact but also potentially through droplets [1]. This highlights the necessity to strengthen case search and screening in Guangzhou.

The MPXV exhibits varying degrees of virulence among its clades, with the strains from the Central African clade (clade I) obviously being more virulent and having higher mortality rates than the West African clade (clade II) [32,33,34]. This difference is attributed to genetic factors: in the West African clade, the absences of some gene fragmentation within open reading frames contribute to reduced virulence, whereas the intact genes in the Central African clade can inhibit T cell activation, suppressing the patient’s immune response [1,35]. Utilizing Nanopore sequencing, we successfully obtained a 195,405 bp length of the complete MPXV clade Ib sequence from the case, designated as hMpxV/China/JXHY/2024/12, with 127 nucleotide alterations and 57 amino acid mutations, which may lead to changes in protein function in organisms. The research on the specific impact of amino acid mutations in the mpox virus is limited. Apart from their role in virus typing, the significance of these mutations in the context of MPXV pathogenesis remains unclear.

Mpox is primarily transmitted through close contact with individuals who are infected with the virus. Historically, mpox infections were associated with contact with infected animals or travel to regions where the disease was endemic [36,37,38]. However, the primary mode of mpox transmission has undergone a significant shift. At present, the majority of cases are related to sexual contact, moving away from the traditional associations with infected animals or travel [39]. With deepening international cooperation and the rising frequency of cross-border personnel exchanges, it is imperative to adopt more vigilant measures for the prevention and control of mpox.

In summary, we reported the genomic analysis of the MPXV clade Ib variant first emerging in China, in order to improve the prevention and control of mpox outbreaks and the monitoring of MPXV mutations.

## Figures and Tables

**Table 1:** Real-time PCR results of various specimens from the imported case.

Specimen	Source	*C_T_*
Herpes swab	Face	26.09
Oropharyngeal swab	Oropharynx	26.50
Herpes swab	Protothorax	27.94
Herpes swab	Abdomen	26.78
Herpes swab	Back	18.63
Herpes swab	Left arm	19.04
Herpes swab	Left buttock	18.10
Herpes swab	Left lower extremity	20.08
Herpes swab	Right arm	18.11
Herpes swab	Right femoral	19.59
Herpes swab	Right lower extremity	19.03
Plasma	Blood	34.08
Full blood	Blood	Negative

**Table 2:** The 57 amino acid mutations of hMpxV/China/JXHY/2024/12.

Position (bp)	Gene	Nucleotide Mutations	Amino Acid Mutations
1368	*OPG001*	257A>C	N86T
3192	*OPG003*	1460C>T	T487I
5377	*OPG015*	745G>A	A249T
5979	*OPG015*	143T>C	I48T
6111	*OPG015*	11T>C	V4A
7900	*OPG019*	334G>T	G112C
11,487	*OPG023*	1583G>A	C528Y
16,320	*OPG027*	237A>C	L79F
21,582	*OPG034*	551C>A	P184H
23,143	*OPG036*	49G>A	V17I
27,161	*OPG040*	480G>A	M160I
28,787	*OPG042*	476C>T	T159I
29,918	*OPG043*	203A>G	N68S
36,113	*OPG052*	29C>G	T10S
36,669	*OPG053*	168G>A	M56I
38,108	*OPG054*	35G>A	R12Q
48,904	*OPG066*	664C>A	L222I
49,148	*OPG066*	420G>T	K140N
53,006	*OPG070*	34T>C	Y12H
73,259	*OPG089*	1009A>G	K337E
78,198	*OPG096*	224T>C	L75P
80,796	*OPG100*	401G>A	R134H
97,180	*OPG115*	124T>C	Y42H
99,175	*OPG117*	730G>A	E244K
103,775	*OPG120*	371C>T	S124L
108,574	*OPG125*	1478C>T	T493M
110,453	*OPG126*	76G>T	A26S
110,647	*OPG127*	577T>C	Y193H
119,766	*OPG136*	2220G>A	M740I
124,029	*OPG140*	116C>A	P39H
126,571	*OPG145*	227C>A	P76Q
130,270	*OPG150*	94G>A	D32N
132,139	*OPG151*	818G>A	R273Q
140,136	*OPG155*	302T>G	I101R
140,573	*OPG156*	783G>A	M261I
145,111	*OPG164*	392A>T	N131I
145,155	*OPG164*	436G>A	V146I
148,457	*OPG170*	286G>A	V96I
150,078	*OPG173*	62A>G	N21S
150,101	*OPG173*	85A>G	I29V
150,383	*OPG174*	994G>A	D332N
150,434	*OPG174*	943C>T	R315C
159,350	*OPG185*	716C>T	T239I
161,996	*OPG188*	680C>T	S227F
165,883	*OPG191*	3A>G	I1M
171,226	*OPG199*	426A>G	I142M
172,116	*OPG200*	155G>A	G52D
179,159	*OPG208*	101T>C	V34A
182,565	*OPG210*	1424C>T	A475V
184,309	*OPG210*	3168G>T	E1056D
185,991	*OPG210*	4850G>A	G1617E
186,727	*OPG210*	5586T>G	D1862E
190,857	*OPG015_dup*	11T>C	V4A
190,989	*OPG015_dup*	143T>C	I48T
191,591	*OPG015_dup*	745G>A	A249T
193,776	*OPG003_dup*	1460C>T	T487I
195,600	*OPG001_dup*	257A>C	N86T

## Data Availability

The 524 reference strains can be queried and were downloaded from NCBI and GISAID, and are accessible at https://doi.org/10.55876/gis8.250323ya. Sequence information on hMpxV/China/JXHY/2024/12 can be obtained upon request from the corresponding author.

## Supplementary Materials

The following supporting information can be downloaded at: https://www.mdpi.com/article/10.3390/pathogens14010102/s1, Table S1: The 127 nucleotide mutations of hMpxV/China/JXHY/2024/12.
